# Allergens with Protease Activity from House Dust Mites

**DOI:** 10.3390/ijms18071368

**Published:** 2017-06-27

**Authors:** Manuel Reithofer, Beatrice Jahn-Schmid

**Affiliations:** Institute of Pathophysiology and Allergy Research, Medical University of Vienna, Vienna 1090, Austria; manuel.reithofer@meduniwien.ac.at

**Keywords:** house dust mite, allergen, protease, Der p 1

## Abstract

Globally, house dust mites (HDM) are one of the main sources of allergens causing Type I allergy, which has a high risk of progressing into a severe disabling disease manifestation such as allergic asthma. The strong protease activities of a number of these allergens are thought to be involved in several steps of the pathophysiology of this allergic disease. It has been a common notion that protease activity may be one of the properties that confers allergenicity to proteins. In this review we summarize and discuss the roles of the different HDM proteases in the development of Type I allergy.

## 1. Introduction

Type I allergy is a hypersensitivity reaction of our immune system, and is mediated by IgE antibodies that recognize otherwise innocuous antigens (usually proteins) from the environment, for instance, pollen or mite allergens. In predisposed individuals, an imbalanced immune response characterized by the excess of CD4 T helper (Th)2 cells leads to the production of elevated levels of allergen-specific IgE antibodies [1]. These Th2 cells represent polarized, differentiated T helper cells, which produce Interleukin (IL)-4, IL-5 and IL-13 as signature cytokines. The Th2 cytokines IL-4 and IL-13 are responsible for the antibody class switch in B cells from IgM or IgG antibodies to IgE [2]. Th2 cells are counter-regulated by Th1 cells via the production of their signature cytokine interferon (IFN)-γ which inhibits the proliferation of Th2 cells [3]. Most of the secreted IgE antibodies stably bind to high-affinity receptors on mast cells or basophils. Upon re-exposure to allergen, receptor bound IgE molecules get cross-linked and activate these effector cells, leading to the immediate release of preformed, inflammatory mediators such as histamine from intracellular granules, which then elicit typical allergic symptoms (hayfever, asthma etc.) [1]. In contrast to seasonal allergens such as pollen allergens, there are allergens that are present the whole year round. The most important source of perennial allergens is house dust mites (HDM). These animals cohabitate with us and are mostly found in bedsheets and mattresses where they feed on skin scales [4]. The most important mite species are *Dermato-phagoides pteronyssinus*, *D. farinae* and *Blomia tropicalis.* Investigations in Central Europe detected major HDM allergens in about 70% of the housings [5]. In recent studies, it was estimated that 65 to 130 million individuals worldwide are sensitized (i.e., possess detectable allergen-specific IgE in their serum) against HDM [6], and in industrialized countries this represents 15–20% of the population [7,8,9]. In Central Europe, 50% of allergic patients are sensitized to dust mites [10]. Patients with house dust mite allergy suffer from various disease manifestations such as allergic rhinoconjunctivitis, atopic dermatitis and, especially, allergic asthma. The latter is characterized by recurrent attacks of breathlessness and wheezing upon inhalation of allergen and is caused by bronchoconstriction and airway inflammation, [4,11,12,13]. About 50% of asthmatic patients are sensitized to HDM allergens [6]. In addition to genetic factors, exposure to house dust mite allergens in early childhood is an important determinant of the subsequent development of asthma [14].

The high prevalence of allergy to HDM instigated extensive research on HDM allergens. A high number of sensitizing proteins and glycoproteins (i.e., causing IgE-production in allergic individuals) have been characterized. The different HDM allergens have been classified into groups containing closely related homologs from different mite species. So far, 20 different protein families have been officially classified as HDM allergens (www.iuis.org) and some more are still under investigation [15]. The major HDM allergens (i.e., allergens recognized by the majority of HDM allergic subjects) are present in high amounts in the fecal pellets of these animals. The pellets become airborne particles that can easily be inhaled, and contain bacterial components stimulating the innate immune system [14]. Group 1 allergens comprise cysteine proteases including the well-studied major allergen Der p 1 (Table 1). Another major allergen is the lipid binding protein Der p 2, which mimics the myeloid differentiation antigen 2 (MD-2), and thus is able to activate innate immune responses [16]. The recently described HDM allergen Der p 23 is also recognized with high prevalence by IgE in allergic subjects. It represents a peritrophin-like protein with homology to chitin binding protein, which—at the moment—has no obvious ascribed functional relevance for the development of allergy [17]. Sensitization to Der p 1 or Der p 2 is found in 80% of mite allergic patients, and 50–60% of their HDM-specific IgE is directed against these two major allergens. Among these HDM allergens, proteases are strikingly abundant. In addition to the group 1 allergens, HDM contain also other allergens with potent proteolytic potential, namely the serine proteases of the group 3, 6 and 9 allergens (Table 1) [18]. The high number of protease allergens present in HDM is thought to contribute to the high allergenic potential of this allergen source.

In this review, we mainly focus on group 1 allergens, represented by the most extensively studied allergen derived from the mite species *Dermatophagoides pteronyssinus*, the major allergen Der p 1 [19]. These papain-like cysteine proteases are endowed with a very high proteolytic potential. In general, allergens can have completely different biological functions, but Der p 1 serves as paradigm for the notion that the allergenicity of a protein is due to its protease activity.

### Group 1 Allergens

**Figure 1:**
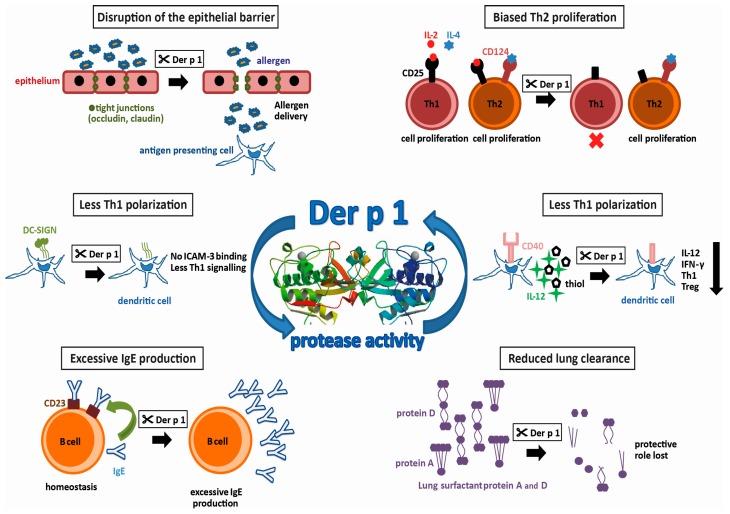
Protease activities possibly promoting allergenicity of Der p 1 (allergen 3D structure, 2AS8 structure [30]).

Group 1 allergens in mites belong to the cysteine protease family and are present in high amounts in the fecal pellets of HDMs, suggesting a biological role in the digestive tract of the mite [20,21]. The first HDM allergen described—and the most intensively studied so far—is Der p 1, a 25 kDa glycoprotein [19]. Actually, it was the first of all allergens to be cloned and expressed as a recombinant allergen [22]. However, recombinant expression of functionally intact Der p 1, which still binds IgE, is difficult. It has to be produced as a pro-enzyme that requires cleavage to become an active enzyme. Most successfully, this has been achieved by the removal of the *N*-glycosylation site and expression in *Pichia pastoris* [23]. In addition to the sequence homology, the X-ray structures documented the similarity to papain. Both contain equally-sized juxtaposed amino- and carboxy-terminal globular domains, forming a cleft embedding the catalytic site. Testing the activity profile of Der p 1 has revealed a distinct substrate specificity which is determined by alanine at the P2 position [24,25]. Further X-ray structures obtained from antibody-bound Der p 1 revealed epitopes for IgE-binding and IgE-crossreactivity with Der f 1 [26,27]. The strong protease activity of Der p 1 has drawn keen interest in allergy research. By comparing antibody production in mice immunized with Der p 1 or Der p 1 with its prototeolytic site irreversibly inhibited, it was shown that the enzymatic activity of Der p 1 has, in fact, an impact on its allergenicity [28]. Using humanized SCID-mice, the group of Lambrecht was able to induce mite-specific Th2 allergic inflammation by intratracheal injection of Der p 1-pulsed, human antigen presenting cells (APC) indicating that Der p 1 alone represents a potent inhalant allergen [29]. In the following paragraphs, we will focus on HDM proteases and the potential molecular mechanisms fostering the development of type I allergy. A summary of these effects resulting from the proteolytic activity of Der p 1 is illustrated in Figure 1.

## 2. Disruption of the Epithelial Barrier

Tight junctions between neighboring mucosal epithelial cells, consisting of the proteins occludin and claudin, regulate the paracellular transport of molecules through the airway mucosa. Normally, they prohibit uncontrolled entry of particles such as pollen or HDM fecal pellets. It has been demonstrated that, by its protease activity, Der p 1 increases the permeability of the airway mucosa by targeting the tight junction proteins [31,32]. This disruption of the epithelial barrier then leads to an increased influx of allergens, to a so called “facilitated allergen delivery” [33]. More recently, it has been shown that exposure of nasal epithelium to Der p 1 leads not only to the cleavage of already present tight junction proteins, but also to a decreased expression of these proteins [34]. In vitro, it has been demonstrated that Der p 1 is able to induce the production of chemokine C-C ligand 2 (CCL2), CCL5 and CXCL10 in bronchial epithelial cells, as well as the subsequent increase of migration of APCs such as Langerhans cells and monocyte-derived dendritic cells into these epithelial layers [35]. Upon allergen-uptake, these cells usually migrate to draining lymph nodes and activate allergen-specific, naïve Th cells (antigen inexperienced, unpolarized T cells). Moreover, Der p 1 has been shown to induce the production of pro-inflammatory cytokines like GM-CSF, IL-6 and IL-8 by airway epithelial cells in vitro [36,37], via activating protease-activated receptor-2 [38]. These cytokines then would lead to extravasation of innate inflammatory cells, such as neutrophils or eosinophils into the tissue in vivo. Thus, all these effects of Der p 1 on the epithelium can promote early processes in allergic sensitization but pathogenic processes in established type I allergy.

## 3. Th2-Biased Immune Response

There are different mechanisms by which the Der p 1-protease activity can contribute to Th2-related disease. One way to do this is the promotion of Th2 responses. As mentioned, Der p 1 can trigger the production of IL-6 by epithelial cells. IL-6 is a cytokine with pleiotropic effects and it has been shown to promote, amongst others, Th2 cell differentiation by inducing early IL-4 expression and, at the same time, inhibition of IFN-γ signaling and consequently Th1 differentiation [39]. In addition, the protease activity of Der p 1 can influence the polarization of CD4 T cells by targeting molecules present on either T cells or APCs. For instance, CD25, the α subunit of the IL-2 receptor, has been shown to be another target of the proteolytic activity of group 1 allergens [40]. In contrast to Th2 cells, which express receptors for the growth factors IL-2 and IL-4, Th1 cells only use IL-2 for expansion and cytokine production. On the other hand, proliferation of Th2 cells is counter-regulated by IFN-γ which is produced by Th1 cells. Therefore, the destruction of the IL-2 receptor by Der p 1 inhibits the proliferation of Th1 cells and the ensuing lack of IFN-γ redounds to the advantage of Th2 helper cells. Recently, using papain as a surrogate for Der p 1, epithelial-derived cytokines such as IL-33, thymal stromal lymphopoietin or IL-25 have been shown to activate type 2 innate lymphoid cells (ILC2), which are particularly present in lung mucosa and represent the earliest source of Th2 cytokines prior to the development of adaptive Th2-cells [41].

## 4. Reduced Th1 Polarization

On the other hand, a reduction of Th1-differentiation also leads to a Th2 bias. The presentation of processed antigen by APCs to T cells is a crucial step in the adaptive immune response. Depending on co-stimulatory molecules present on the surface of the APCs and the local cytokine milieu, the differentiation of naïve T cells is either driven towards Th1- or Th2 effector cells. An important costimulatory molecule on APC is CD40. Binding of CD40 to its ligand CD40L on naïve T cells, leads not only to increased production of both Th1 and Th2 cytokines, but also to the production of IL-12 by the APC, which induces the differentiation of Th1 cells [42]. In vitro, it has been demonstrated that in the presence of Der p 1, CD40 is cleaved, and the resulting lack of IL-12 results in both a lower production of IFN-γ and an increased production of IL-4 [43]. The resulting cytokine milieu again favors the polarization of naïve T cells into Th2 cells. In addition, the ligation of CD40 on APC affects another factor of Th1-promotion, namely the production of extracellular thiols. It has been shown that the presence of Der p 1 decreases the production of thiols in vitro, and thus supports a Th2-biased immune response [44]. Concerning another co-stimulatory molecule involved in Th polarization, an in silico approach using a prediction tool for protease specificity (PoPS) revealed further immune relevant substrates of Der p 1. As a result, the cleavage of dendritic cell-specific ICAM-grabbing non-integrin (DC-SIGN) and the DC-SIGN receptor on APCs by group 1 allergens has been suggested [25]. Investigation of the predicted targets in vitro actually revealed that the cleavage by Der p 1 reduced the interaction of APCs with naïve T cells via intracellular adhesion molecule-3 (ICAM-3). As ICAM-3 is thought to be involved in preferential activation of Th1 signaling, it was suggested that the cleavage of DC-SIGN by Der p 1 again facilitates Th2-differentiation. Together, the promotion of Th2 cells leading to the unbalanced T cell response to allergens is considered as a major factor underlying the high allergenic potential of the group 1 mite allergens [40,45,46].

## 5. Reduced Lung Clearance

Pulmonary surfactant, a lipoprotein complex secreted by airway epithelial cells, plays a role in host defense. SP-A and SP-D, are two lung surfactant proteins with crucial functions in innate immune responses by opsonizing pathogens and facilitating their uptake by phagocytosis into innate immune cells [47]. Pulmonary surfactant may also be important for allergic diseases with regard to the clearance of allergens. It has been shown that pulmonary surfactant is capable of binding allergens, and therefore may reduce allergic sensitization by allergen removal or interference with IgE-binding and thus prohibit allergic reactions [48,49]. Studies in mouse models of allergy have shown therapeutic effects by the administration of lung surfactant proteins [50,51]. It was found more recently that Der p 1 is able to cleave SP-A and SP-D [52]. This capability to eliminate the protective pulmonary surfactants may be another cause for the high allergenicity of group 1 allergens by reducing their protective effects.

## 6. Excessive IgE Production

The level of IgE-production by B cells is regulated by a negative feedback mechanism that involves IgE-binding to CD23, the low-affinity receptor for IgE (FcεRII), which is present on the surface of these cells. Upon binding of IgE/allergen-complexes to CD23, B cells downregulate their production of IgE [53,54]. It has been shown in vitro that Der p 1 is able to disrupt this IgE-feedback mechanism by selective cleavage of CD23 [55,56]. Consequently, affected B cells produce excessive amounts of IgE, and the homeostasis of IgE is lost [45,55,56]. A disruption of this negative feedback loop for IgE-production in B cells eventually results in the progression of the allergic disease, and thus is another factor that contributes to the high allergenicity of the group 1 mite allergens.

### Group 3, 6 and 9 Allergens

In addition to the family of papain-like cysteine proteases, a “triad” of serine proteases, comprising trypsin-like, chymotrypsin-like and collagenolytic-like proteases, is also present in fecal pellets of HDM [18] (Table 1). The cDNAs of these proteases have also been cloned and sequenced in the most important mite species [6]. These allergens are much less abundant, as the group 1 allergens in HDM extracts and IgE specific to these molecules is less prevalent in patients with allergy to HDM, and therefore have not been studied as intensively as the group 1 allergens [57,58,59,60]. Several studies have indicated that in principle their protease activity is similar to that of group 1 allergens, for instance regarding their effects on the epithelial barrier [57]. Group 3, 6 and 9 proteases are also able to cleave the transmembrane protein occludin of tight junctions, increase the permeability of the epithelial barrier, and cause the release of pro-inflammatory cytokines [36,61]. As for Der p 1, the proteinase-activated receptor 2 was also defined as target for these proteases. Activation of this receptor induces the expression and release of pro-inflammatory cytokines or chemokines like IL-8 or eotaxin and attraction of innate immune cells [37,62,63]. Interestingly, a recent report demonstrated that Der p 1 is actually the primary activator of the serine proteases from *Dermatophagoides pteronyssinus* by cleaving the respective pro-enzymes. Thus, first of all, Der p 1 is needed for the maturation of these serine proteases [64,65,66]. Since Der p 1 is also much more abundantly present in house dust than the serine proteases, it can be assumed that its activity plays a much greater role in the pathomechanisms involved in the development of type I allergy to HDM than the group 3, 6 and 9 serine proteases.

## 7. Conclusions

IgE-mediated allergy to HDM allergens represents a considerable health burden, as it is one of the major causes for asthma in industrialized countries. HDM allergens include several proteases with strong proteolytic activity, which can contribute to the allergic sensitization process as well as to the progression of the disease. HDM proteases cleave different substrates that are involved in diverse immune mechanisms starting from epithelial barrier function, proceeding to innate inflammatory responses, involving T cell polarization, and even have a direct impact on IgE-production by B cells. However, for many substrates of these proteases, the identified mechanisms have only been studied in vitro, and still need further investigations to prove their actual relevance in vivo. Most recently, the evaluation of the evolutionary pattern of house dust mite sensitization in a longitudinal birth cohort study by chip technology has revealed that Der p 1 is not only one of 3 HDM allergens that characterize early sensitization to HDM, but (together with Der p 23) is also significantly associated with subsequent asthma development that usually starts at school age [67]. This observation may support the assumption that group 1 protease allergens play a pivotal role in the development of IgE-mediated allergy and especially allergic asthma. However, it has been debated recently that the allergenicity of group 1 allergens is rather due to exposure to particles containing high amounts of allergen in combination with immune-stimulatory molecules than to its intrinsic proteolytic activity [68].

## Figures and Tables

**Table 1:** Protease allergens in house dust mites. Adapted from Moises A. Calderón, et al. [6].

Allergen Group	Identified Allergens	Protein Family	Prevalence
1	Der p 1, Der f 1, Der m 1, Der s 1, Eur m 1, Blo t 1, Pso o 1, Sar s 1	Cysteine protease, papain-like	80%
3	Der p 3, Der f 3, Der s 3, Eur m 3, Blo t 3, Sar s 3, Gly d 3, Lep d 3	Trypsin-like serine protease	16–100%
6	Der p 6, Der f 6, Blo t 6	Chymotrypsin-like serine protease	40%
9	Der p 9, Der f 9, Blo t 9	Collagenolytic-like serine protease	90%

Der p, *Dermatophagoides pteronyssinus*; Der f, *D. farinae*; Der m, *D. microcera*; Der s, *D. siboney*; Eur m, *Euroglyphus maynei*; Blo t, *Blomia tropicalis*; Pso o, *Psoroptes ovis*; Sar s, *Sarcoptes scabie*; Lep d, *Lepidoglyphus destructor.*
